# Phylogenetic signal in flowering phenology weakens over elevation in the high Andes of Chile: evidence for evolutionary convergence in a harsh habitat

**DOI:** 10.3389/fpls.2026.1738754

**Published:** 2026-02-17

**Authors:** Ítalo Tamburrino, Valeria Robles, Paola Jara-Arancio, Pablo C. Guerrero, Jesús López-Angulo, Jeannine Cavender-Bares, Mary T. K. Arroyo

**Affiliations:** 1 Departamento de Ciencias Ecológicas, Facultad de Ciencias, Universidad de Chile, Santiago, Chile; 2 Centro Internacional Cabo de Hornos (CHIC), Universidad de Magallanes, Punta Arenas, Chile; 3 Instituto de Ecología y Biodiversidad (IEB), Concepción, Chile; 4 Departamento de Ciencias Biológicas y Departamento de Ecología y Biodiversidad, Facultad de Ciencias de la Vida, Universidad Andrés Bello, Santiago, Chile; 5 Departamento de Botánica, Facultad de Ciencias Naturales y Oceanográficas, Universidad de Concepción, Concepción, Chile; 6 Millennium Institute Biodiversity of Antarctic and Subantarctic Ecosystems (BASE), Santiago, Chile; 7 Instituto de Investigación en Cambio Global (IICG-URJC), Universidad Rey Juan Carlos, Móstoles, Spain; 8 Departamento de Biología y Geología, Física y Química Inorgánica, Universidad Rey Juan Carlos (URJC), Móstoles, Spain; 9 Harvard University Herbaria and Department of Organismic and Evolutionary Biology, Harvard University, Cambridge, MA, United States; 10 Department of Ecology, Evolution, and Behavior, University of Minnesota, Saint Paul, MN, United States

**Keywords:** alpine plants, Andes, elevation gradient, flowering phenology, functional diversity, phylogenetic signal, phylogenetic structure

## Abstract

**Introduction:**

High elevation plants experience cold temperatures and short growing seasons that constrain their flowering window. These environmental limitations are expected to promote strong overlap in flowering phenology among co-occurring species. Whether similarity in flowering times arises from environmental filtering of lineages preadapted to cold conditions or from evolutionary convergence in response to shared selective pressures remains unclear. We hypothesize that flowering phenology of high alpine communities is the result of convergence due to strong selective pressure imposed by the environment rather than environmental filtering for conserved ancestral traits.

**Methods:**

To test this hypothesis, we analyzed the functional and phylogenetic structure of phenological traits, as well as their phylogenetic signal, using a molecular phylogeny across four sites spanning subalpine to high alpine zones in the central Chilean Andes. A total of 86 species were included. Observed patterns were compared against null model expectations to evaluate functional convergence, phylogenetic structure, and trait conservatism.

**Results:**

High alpine sites exhibited earlier flowering following snowmelt, greater functional convergence in phenological traits, and significant phylogenetic signal in a smaller subset of floral traits compared to subalpine sites. Additionally, both high alpine communities showed significant terminal phylogenetic clustering.

**Discussion:**

Our results suggest that environmental filtering of preadapted lineages plays a minor role in shaping high alpine community structure. Rather, intense environmental pressures at higher elevations appear to drive trait convergence in flowering phenology. We conclude that the general trend for phylogenetic conservatism in floral phenology has been overridden by the harsh environmental conditions in the high Andes. The high alpine environment can be seen as an evolutionary promoter of convergent phenological strategies rather than a gatekeeper of lineages preadapted to cold conditions.

## 1 Introduction

Species composition in communities reflects species-specific tolerance and performance under the multiple challenges posed by both their physical and biological environments (Baraloto et al., 2012). Because such tolerance depends on functional traits (Violle et al., 2007; Caruso et al., 2020), these traits define the taxonomic composition of any community by setting the boundaries of tolerable conditions (Krishna et al., 2021). Nonetheless, while some traits are conserved and remain functional across various abiotic and ecological contexts, others are likely to change over evolutionary timescales due to adaptive processes (Burns and Strauss, 2012). For example, certain floral structures, such as the presence of two fused thecae and an outer integument in the ovule, are widespread across plants, whereas traits such as the number of floral parts, sexual expression (e.g., hermaphroditism), and flower shape are highly variable (Bateman et al., 2006).

Numerous studies have shown that traits of closely related species tend to resemble each other more than those of distantly related species (Davies et al., 2013). At the same time, closely related species tend to remain in the same biome (Crisp et al., 2009). These patterns assume that members within lineages inherit trait states from their common ancestors and that traits evolve through small and random changes due to genetic drift, resulting in variation proportional to divergence times (Blomberg and Garland, 2002). A close relationship between phylogenetic distance among taxa and trait similarity indicates a strong phylogenetic signal. Conversely, when similar traits occur in distantly related taxa or are randomly distributed across the phylogeny, phylogenetic signal is weakened. In such cases, evolutionary forces such as adaptation may have played a significant role.

Nonetheless, there is disagreement regarding whether phylogenetic signal in biological traits is ubiquitous or if it is found only under certain conditions. Some authors argue that ecological niches are highly conserved due to stabilizing selection and gene flow, resulting in strong phylogenetic signal in ecologically relevant traits (Ackerly, 2004; Wiens and Donoghue, 2004; Johnson and Stinchcombe, 2007). To the contrary, others claim that it only occurs in some clades and not in all traits (Kembel and Hubbell, 2006; Losos, 2008). In this sense, there are many reasons why functional traits should show evolutionary lability (Pearman et al., 2008). Classic niche theory indicates that coexistence among species exploiting the same resources favours trait divergence, thereby avoiding competition (Macarthur and Levins, 1967; Vellend, 2006; Costa-Pereira et al., 2019). On the other hand, species sharing a single and scarce resource will inevitably converge in traits (Fox and Vasseur, 2008). Besides the existence of shared resources, shared environmental conditions should also lead to trait convergence (Cavender-Bares et al., 2004; Givnish, 2016), especially when environmental conditions are extreme (Losos, 2011). Nevertheless, no clear pattern currently exists regarding the physical environmental conditions under which traits show greater or weaker phylogenetic signal.

Strong conservatism or lability influence community assembly by shaping the distribution of species within the functional trait space, hereafter referred to as ‘functional structure’. This functional structure interacts with processes such as limiting similarity (i.e. competitive exclusion among ecologically similar species, Webb et al., 2002), and environmental filtering, defined as biotic and abiotic conditions that allow certain taxa to establish and persist (Kraft et al., 2015). Environmental filtering and dispersal history at different timescales determine the occurrence of lineages across habitats (Cai et al., 2024). Webb et al. (2002) provided a succinct framework to describe contrasting assembly outcomes under different scenarios of trait conservation or divergence: (1) environmental filtering acting over a conserved trait leads to phylogenetically clustered communities, as only taxa from lineages with certain traits can exist in particular habitats; (2) environmental filtering acting on a labile trait leads to phylogenetically dispersed communities composed of members from different lineages; (3) limiting similarity acting on a conserved trait results in phylogenetic overdispersion, as taxa exclude their most similar relatives; and (4) limiting similarity acting on a labile trait can produce even or random patterns in phylogenetic structure, as the phylogenetic position of the more similar taxa is not fixed. While this framework has proved useful and has been further refined and extended (Kraft et al., 2007; Cavender-Bares et al., 2009; Vamosi et al., 2009), the community assembly process might not be so straightforward (Gerhold et al., 2015; Münkemüller et al., 2020). Studies in a greater diversity of communities characterized by different environmental characteristics and the consideration of a broader range of functional traits are required to reach a better understanding.

The alpine gradient provides an ideal scenario for exploring the interplay between abiotic factors, phylogenetic effects on functional traits and community structure. Temperatures decrease with increasing elevation at an average global rate of 6 °C per 1000 m elevation, snowmelt occurs progressively later, and the growing season becomes shorter with increasing elevation (Körner, 2021). These changes take place over short spatial distances and where photoperiod shows little variation. In general, not all individuals in a population flower at the same time, suggesting room for selective adjustment of flowering time within species (Forrest and Miller-Rushing, 2010). Recent studies over elevational gradients have detected flowering phenology adjustment among populations of the same species expressed in progressively earlier flowering and lower thermal sums required to trigger flowering at higher elevations (Bucher et al., 2018; Arroyo et al., 2021). It has been suggested that such adjustments optimize the use of the growing season and increase fecundity, providing that they do not produce mismatches with pollinators (Sparks et al., 2000; Galvagno et al., 2013; Baeten et al., 2015). If phenological adjustment occurs within populations of the same species, a parallel situation could be expected comparing flowering times for the sets of species found at different elevations above treeline —i.e., at the community level. This could lead to lineages shifting their flowering dates in unison to the extent that phylogenetic signal remains unchanged over elevation. In this context, several studies have shown that the temporal sequence of flowering among species in the season is characterized by strong phylogenetic signal at both the community (Staggemeier et al., 2010; Lessard-Therrien et al., 2014; Du et al., 2015) and global level (Davies et al., 2013). Nevertheless, under the shorter growing season and stronger climatic constraints at higher elevations, there is some evidence for convergence in flowering times at the community level and a weakening of phylogenetic signal (Lessard-Therrien et al., 2014). The existence of phylogenetic conservatism in flower phenology yet strong environment constraints at high elevations provides an ideal situation for disentangling whether phylogenetic signal weakens under such conditions and if high elevation communities have been shaped by environmental filtering for lineages capable of thriving in the short growing season or by trait convergence occurring in distantly related taxa.

Here, we investigate the functional and phylogenetic structure, as well as phylogenetic signal of flowering phenology, in plant communities in two high elevation vegetation belts in the central Andes of Chile to untangle the processes underlying community assembly. Specifically, we aim (1) to test whether plant communities at the high alpine belt show differences in flowering times compared with to communities in the lower subalpine belt and (2) to determine whether these changes reflect environmental filtering of lineages or convergent evolution in their flowering times. We hypothesize that communities at higher elevations have experienced stronger convergent evolution due to the harsher environmental conditions and are not necessarily the result of environmental filtering. We expect to find greater convergence in flowering phenology traits and lower values of phylogenetic signal in the high alpine communities compared with their subalpine counterparts. Under these circumstances, basal phylogenetic clustering, expected under environmental filtering, should not be observed in the high alpine.

## 2 Materials and methods

### 2.1 Study area

Work was carried out in the Andes east of Santiago, in the Farellones-La Parva-Valle Nevado area (~33° S). This area of the Andes has a semi-arid climate with a Mediterranean influence. Snow begins to accumulate from April-June and remains on the ground until late August-September to late November depending on elevation, with considerable variation among years. The climate during the spring and summer months is mainly sunny. However, cloudy days, intermittent afternoon cloudiness and windiness increase with elevation (Viale and Garreaud, 2014) and short-duration summer snow and hail may be received. January and February are warm months (Valle Olivares weather station and El Yeso Embalse, 33.68°S, 70.09°W, 2475 m.a.s.l. - http://explorador.cr2.cl/). Because of the altitudinally depressed treeline caused by aridity (Piper et al., 2016), above treeline gradients in the central Chile Andes extend for over 1000 m elevation providing an ideal system for investigating the questions of interest.

Above treeline vegetation in the central Chilean Andes comprises two physiognomically-recognizable vegetation belts. The first is a subalpine scrub dominated by small, rounded shrubs, accompanied by subshrubs, and perennial and annual herbs (Cavieres et al., 2000). The subalpine scrub belt is superseded by the high alpine belt comprised of flat cushion species, accompanied by subshrubs and perennial herbs, giving way to scattered perennial herbs in its upper limit. In terms of their floristic composition, the distributions of many of the species in these two vegetation belts are limited to the mediterranean-type climate area of central Chile. Nevertheless, the high alpine belt contains a number of species that are distributed widely along the Andean corridor (e.g. *Caltha appendiculata*, *Colobanthus* spp., *Perezia pilifera*, *Azorella monantha*). Northern-hemisphere clades with important presence in the Southern Andes typically involved initial colonization of lowland areas followed by subsequent ascent into alpine habitats (e.g. *Valeriana*, Bell et al., 2012; and probably *Astragalus*, Scherson et al., 2008).

**Figure 1:**
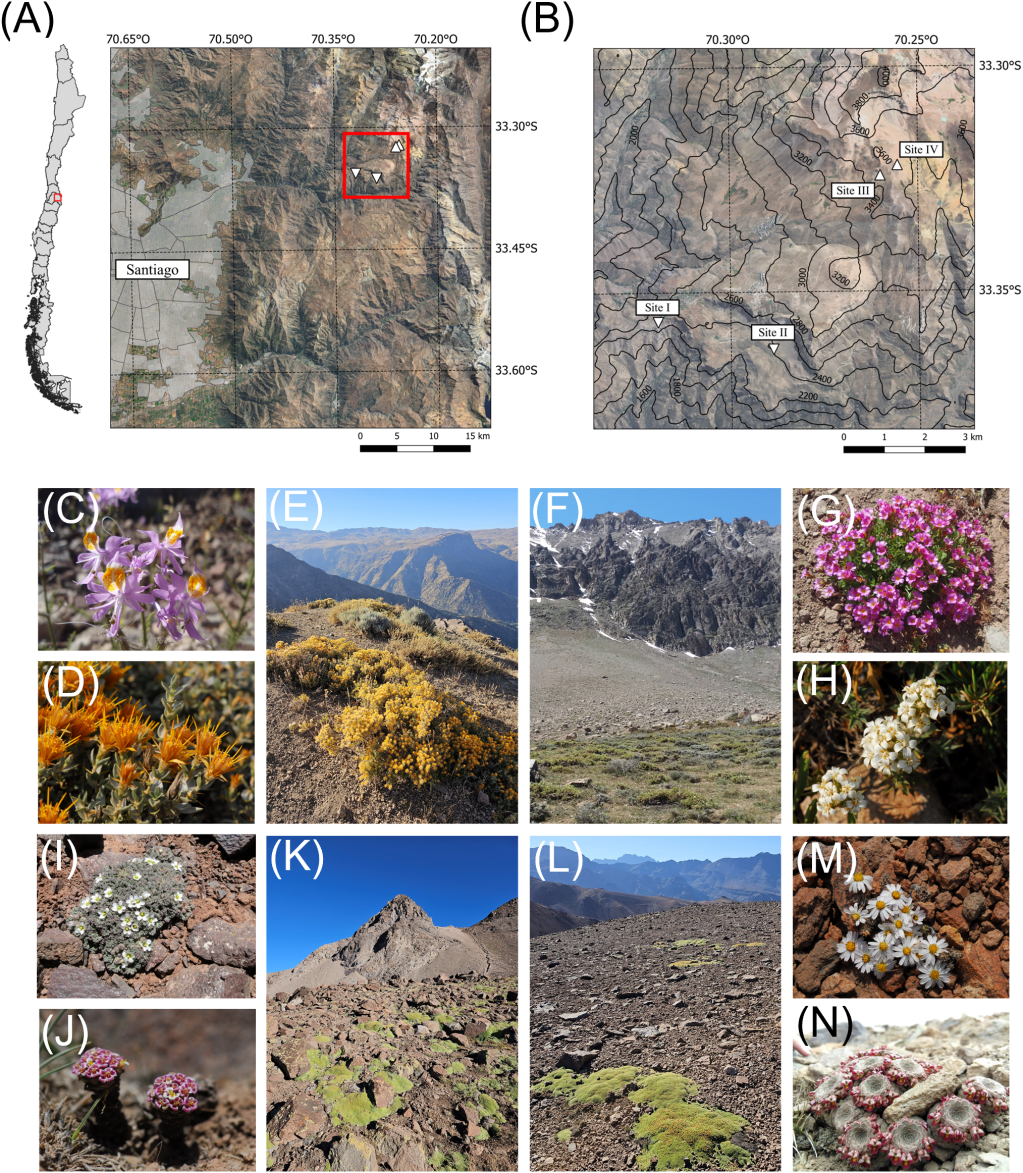
**(A)** Location of study area in central Chile and **(B)** contour-based elevation map of the study area. Sites I **(E)** and II **(F)** are found on different slopes at 2320 masl and 2405 masl in the middle part of the subalpine belt, with mean annual temperature around 7.8 °C (Cavieres et al., 2000). Sites III **(K)** and IV **(L)** are located on different slopes at 3447 masl and 3553 masl in the alpine belt, with mean annual temperature around 1.2 °C (Cavieres et al., 2000). **(C)***Schizanthus hookeri*, **(D)***Chuquiraga oppositifolia*, **(G)***Oxalis squamata*, **(H)***Nassauvia aculeata*, **(I)***Nototriche compacta*, **(J)***Nassauvia lagascae*, **(M)***Oriastrum lycopodioides*, **(N)***Viola atropurpurea*. Photographs C to M by MTKA and photograph N by VR.

Two sites were selected in the zonal vegetation in the subalpine belt (Sites I and II) and two in the high alpine belt (Sites III and IV) (**Figure 1**; see **Table 1** for site characteristics and species numbers). Sites I and III, and Sites II and IV, respectively, pertain to two adjacent but distinct valley systems, with the former predominantly west-facing and the latter mainly east-facing. In the study area, plant species composition changes gradually with increasing elevation (Tamburrino et al., 2025) with only three species shared between our subalpine and high alpine sites. On each site, 10 individuals were marked per plant species. The total numbers of species per site ranged from 20 to 36, with fewer species on the two upper alpine sites due to the lower species richness there. Many species were shared among the two upper and two lower sites, respectively (**Table 1**, see **Supplementary Figure 1** for species composition on each site). A few rare species, where 10 individuals could not be found, were not included. The total number of species considered in the study was 86. Because species in the upper alpine are mostly perennial and tend to begin to flower shortly after snowmelt, all plants (except for a single annual species) were marked in late autumn prior to winter snowfall. Many species in the subalpine sites were likewise marked in the previous autumn. On Site II, individuals in a dense patch of grasses could not be reliably marked as to their species identity and thus were excluded. Where needed, plants were protected with large wire netting enclosures to prevent damage by wandering cattle. As of the date of snowmelt, the sites were visited at five-day intervals until the end of the flowering season, from September 11^th^, 2018, to May 15^th^, 2019, spanning a continuous period of 35 weeks. On each observation date, all individuals of a species that were in flower were registered and the number of open flowers per plant counted. For plants with a very large number of flowers, the number of open flower-bearing branches was counted. For large flat cushion plants, flowering in 10 cm x 10 cm fixed quadrats running across the diameter of the cushion was recorded. For annual species where the density of individuals was very high, quadrats were used to quantify flowering. Snowmelt dates and the duration of phenological observations for each site can be found in **Table 1**.

**Table 1:** Decimal coordinates, elevation, area, species number (N), date of snowmelt, date when first flower opened in the community (FF) and mean date of first flower opening in each species (Mean FF) for each study site.

Site	Latitude	Longitude	Elevation	Area (m^2^)	N	Snowmelt	FF	Mean FF
I	-33.3565	-70.3192	2320 masl	4000	36	Sept. 11th, 2018	Sept. 11, 2018	Nov. 21, 2018
II	-33.3628	-702887	2405 masl	7500	33	Sept. 26th, 2018	October 1, 2018	Dec. 11, 2018
III	-33.3242	-70.26	3447 masl	7000	20	Nov. 11th, 2018	Nov. 11, 2018	Dec. 31, 2018
IV	-33.3219	-70.2554	3553 masl	10000	23	Nov. 6th, 2018	Nov. 6, 2018	Dec. 27, 2018

### 2.2 Flowering metrics and abiotic data

Five calendar-based floral traits and six thermal sum-based traits were defined, giving a total of 11 flowering traits (see **Box 1** for full detail of abbreviations). The calendar-based traits were:

Box 1 **Abbreviations**

**Table:** 

FF_DOY_	Calendar Day Of Year to First Flowering
FF	Days from snowmelt to First Flowering
PFP	Days from snowmelt to Peak number of Flowering Plants
PFA	Days from snowmelt to Peak number of Floral Abundance
FSL	Flowering Season Length
T_BASE_	Base Temperature
GDD_0_FF	Growing Degree Days to FF with T_BASE_ = 0 °C
GDD_5_FF	Growing Degree Days to FF with T_BASE_ = 5 °C
GDD_0_PFP	Growing Degree Days to PFP with T_BASE_ = 0 °C
GDD_5_PFP	Growing Degree Days to PFP with T_BASE_ = 5 °C
GDD_0_PFA	Growing Degree Days to PFA with T_BASE_ = 0 °C
GDD_5_PFA	Growing Degree Days to PFA with T_BASE_ = 5 °C
SES	Standardized Effect Size
FRic	Functional Richness
FDis	Functional Dispersion
RaoQ	Rao’s Quadratic Entropy
MPD	Mean Pairwise Distance
MNTD	Mean Nearest Taxon Distance

1. Day of year when the first individual of a species flowered (FF_DOY_).
2. Days from snowmelt to the first flowering individual per species (FF).
3. Peak flowering plants (PFP), expressed as days from snowmelt to maximum number of plants in flower.
4. Peak floral abundance (PFA), expressed as days from snowmelt to the maximum number of open flowers.
5. Flowering season length (FSL).

Thermal sum-based traits were expressed in Growing Degree Days (GDD) which give the accumulated heat required for achieving reproductive milestones (FF, PPF and PFA). The biofix day was the date of snowmelt (**Table 1**). Snowmelt dates were established by direct observation over the last days the snow lay on the ground. Following other alpine studies, GDDs were calculated for two values of base temperature (T_BASE_), T_BASE_ = 5 ^°^C and T_BASE_ = 0 ^°^C (GDD_0_ and GDD_5_) using hourly temperatures recorded at 1.5 m a.g.l HOBO U23 Pro v2 battery-powered sensors (Onset Computer Corp., Cape Cod, MA, USA). Sensors were protected with custom-made solar radiation shields. The two measures of GDD were calculated with HOBOware using the double sine method with no upper threshold. Calculation of GDD for the aforementioned reproductive milestones resulted in six GDD-based traits (GDD_0_FF; GDD_5_FF; GDD_0_PFP; GDD_5_PFP; GDD_0_PFA and GDD_5_PFA).

Flowering overlap among species on each site was estimated using Augspurger’s flowering synchrony index (Augspurger, 1983) with the *flower* R Package (Michalski and Durka, 2007). This index depicts the degree of overlap between all pairs of plants species on a site based on presence or absence of flowering on each observation date. All calendar-based and GDD-based traits, as well as Augspurger’s index, were compared among sites with ANOVA or the Kruskal-Wallis test, upon normality check. *Post hoc* Tukey test and pairwise Wilcoxon test were used, respectively, to detect differences among pairs of sites. All statistical analyses were carried out in R v.4.1.2 (R Core Team, 2021; RStudio Team, 2023).

### 2.3 Functional structure

Functional structure, i.e. the distribution of traits among species within a community, was estimated using functional diversity indices, calculated for each community from a trait matrix containing locally measured phenological traits for each species. To avoid collinearity among traits and to ensure that diversity metrics reflect orthogonal functional axes, a Principal Component Analysis (PCA) was performed on the standardized trait matrix. The first three principal components were retained, which together accounted for over 98% of the total variance. This dimensionality reduction allows for a more robust and interpretable representation of functional space, while minimizing numerical issues associated with calculating multidimensional convex hulls (Laliberte and Legendre, 2010). The functional structure indices calculated were:

1. FRic (Functional Richness): the volume of the convex hull occupied by species in trait space.
2. FDis (Functional Dispersion): the mean distance of species to the functional centroid.
3. RaoQ (Rao’s Quadratic Entropy): the mean pairwise distance between species.

To assess whether the observed values of the three metrics were greater or lower than expected by chance, functional diversity indices were compared to 9,999 randomized communities generated by randomly selecting species from the species pool of all four sites combined, maintaining species richness. The standardized effect size of the functional structure indices (χ_SES_) was calculated as χ_obs_- χ_null_/SD_null_, where χ_obs_ was the observed functional diversity index, χ_null_ was the mean value of the 9,999 randomizations and SD_null_ was the standard deviation of these simulated values. Additionally, empirical two-tailed p-values were computed as the proportion of null values with equal or greater deviation from the null mean than the observed value. This allowed us to determine whether each community exhibited significantly higher or lower functional structure than expected under random assembly. All calculations were carried out using the *FD* R package (Laliberté et al., 2014).

### 2.4 Phylogenetic inference

To obtain real branch lengths resolved at the species level, a phylogenetic tree based on three genetic markers (ITS, *rbc*L and *mat*K) was constructed. For species where DNA sequences were not already available in GenBank, DNA was extracted in the laboratory from leaf material collected in the field and from herbarium material stored at CONC (Herbarium of the Department of Botany, University of Concepcion) and at SGO (Herbarium of the National Museum of Natural History). Samples collected in the field were stored in silica gel. Vouchers for field-collected material are deposited in the herbaria CONC. *Ginkgo biloba* (Ginkgoaceae) was chosen as outgroup. Downloaded and original sequences are detailed **Supplementary Table 1**.

A concatenation of nuclear marker ITS and plastidial regions *rbc*L and *mat*K (**Supplementary Data**, **Supplementary Table 2** for laboratory protocols and primers details) was used in a Bayesian inference analysis performed in the CIPRES Science Gateway V. 3.3 Portal (www.phylo.org). Three partitions were used corresponding to each gene, in which evolutionary models for each genetic region were: ITS GTR+I+G, *rbc*L GTR+I+G y *mat*K GTR+G were applied based on the Akaike Information Criterion. Parameters were sampled 20 x 10^6^ generations and 25% of the first samples were discarded. Nodes with ≥ 0.95 were considered robust for posterior probabilities (Ronquist et al., 2012). The Tracer program v1.7 (Rambaut et al., 2018) was used to visualize output parameters to prove stationarity and assess convergence of duplicated runs on the same mean likelihood.

### 2.5 Evolutionary model and phylogenetic signal

Phylogenetic signal for the 11 flowering traits was evaluated using Blomberg’s K (Blomberg et al., 2003). Blomberg’s K quantifies the observed similarity among phylogenetically related species relative to expectations under a white noise model (i.e., no phylogenetic signal). A value of K = 1 corresponds to Brownian motion expectation, values less than 1 indicate weaker phylogenetic signal (traits less similar among relatives than expected), whereas values greater than 1 suggest stronger-than-expected trait conservatism within clades. Phylogenetic signal was calculated individually for each site. Regardless of whether traits exhibited a phylogenetic signal, Brownian motion (BM), Ornstein–Uhlenbeck (OU), and early-burst (EB) evolutionary models were fitted to the trait data, and the best model was selected based on the Akaike Information Criterion (AIC). All analyses were performed using the *phytools* and *geiger* package in R (Pennell et al., 2014; Revell, 2024). Given the high consistency between GDD_0_ and GDD_5_ traits, only GDD_0_ results are presented in the main text.

### 2.6 Richness effect assessment and phylogenetic signal

Species richness generally declines monotonically along elevational gradients in high mountains (Körner, 2021), signifying that local communities at higher elevations will naturally tend to contain smaller numbers of species. Münkemüller et al. (2012) concluded that the uncertainty of detecting phylogenetic signal decreases as sample size increases. To rigorously determine whether high elevation communities have been shaped by environmental filtering or trait convergence, the effect of differences in species richness must be dealt with. In this paper, a novel procedure was employed to detect any effect of species richness on the detection of phylogenetic signal and take concrete measures to overcome the large differences in species numbers between our subalpine and high alpine sites. To test for the effect of sample size on phylogenetic signal, 999 random subsamples of species for each site were run, ranging from the original species richness values down to 20 in the subalpine sites and from the original species richness down to 10 in the high alpine sites. Blomberg’s K and associated p-values were calculated and graphed against simulated richness. To overcome the spurious effect of the smaller sample sizes on the upper sites, an artificial single community was constructed by merging the phenology data for the two upper alpine sites. As not all species on the two sites are the same, this provided us with a community with a larger number of species (N = 30) which came close to sample sizes in the two subalpine communities. For the enlarged community, phylogenetic signal was recalculated for all eleven floral traits using mean dates for the shared species. The same procedure of constructing a new single community was carried out also for the subalpine sites to assure that the original results on phylogenetic signal continued to be detected after the merging procedure.

### 2.7 Phylogenetic structure

To investigate possible phylogenetic structure that would be suggestive of the existence of environmental filtering, mean pairwise phylogenetic distance (MPD) and mean nearest taxon phylogenetic distance (MNTD) among species was calculated (Webb et al., 2002) for each site. To test whether the phylogenetic structure of each site differed from random expectation, standardized effect size of MPD and MNTD was calculated using ses.mpd and ses.mntd functions of *picante* R package (Kembel et al., 2010). Positive values of MPD_SES_ or MNTD_SES_ are found when a community has higher observed phylogenetic distances than expected by chance, indicating phylogenetic evenness or overdispersion, whereas negative values are associated with lower phylogenetic distances among taxa, suggesting phylogenetic clustering. MPD is generally thought to be more sensitive to tree-wide patterns of phylogenetic clustering and evenness, while MNTD is more sensitive to patterns of evenness and clustering closer to the tips of the phylogeny (Webb et al., 2002).

## 3 Results

### 3.1 Phylogenetic construction

The total evidence matrix for the 86 species included 5009 nucleotide characters (1103 ITS, 1669 *rbc*L and 2237 *matK*). The topology of the Bayesian Inference tree shows the relationships between the species considered in the study. Most taxa are well supported (>0.95; **Supplementary Figure 2**) and fall into their respective orders and families. The effective sample size (ESS) value was greater than 200 in a range between 2546 and 30945, indicating that the three genetic regions could be legitimately combined.

### 3.2 Communities overview and phenological traits at different elevations

Snowmelt occurred in September on the lowest sites I and II and in November in the highest sites III and IV (see **Table 1** for details). Whereas the snowmelt dates between the subalpine and high alpine sites differed by almost two months, mean date of first flowering, despite the much colder conditions, differed only by one month (November 30^th^*vs* December 29^th^). A few species (*Barneoudia chilensis* in Site I and *Viola atropurpurea* in Sites III and IV) began flowering immediately after snowmelt, whereas in Site II flowering began one week later.

**Figure 2:**
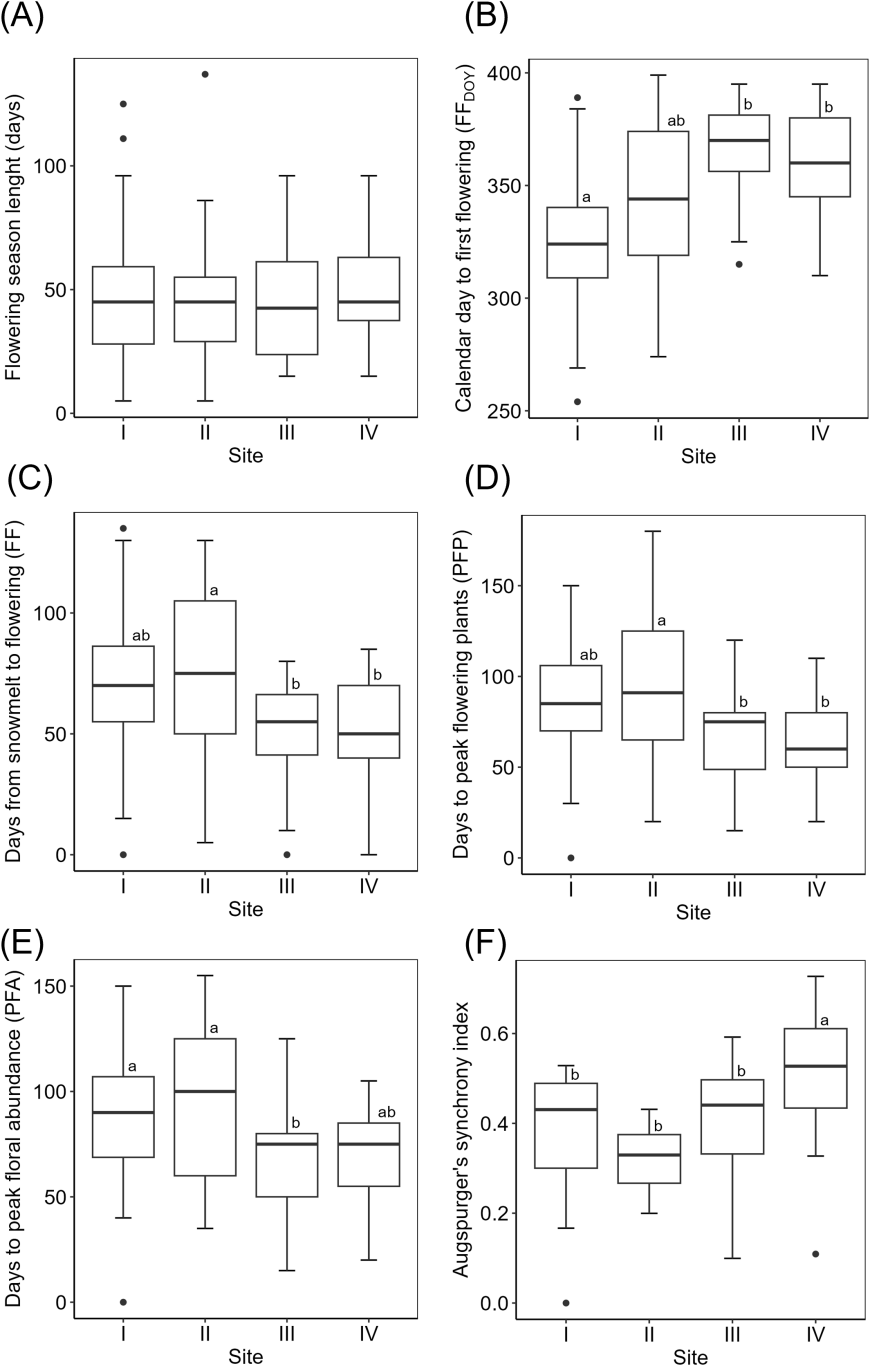
Boxplots of calendar-based phenological traits across four sites. **(A)** Flowering season length; **(B)** day of year of first flowering; days from snowmelt to **(C)** first flowering, **(D)** maximum number of flowering plants and **(E)** maximum number of open flowers; and **(F)** Augspurger's synchrony index. Significant differences between sites based on Tukey test or Wilcoxon test are indicated in lower case letters.

**Figure 3:**
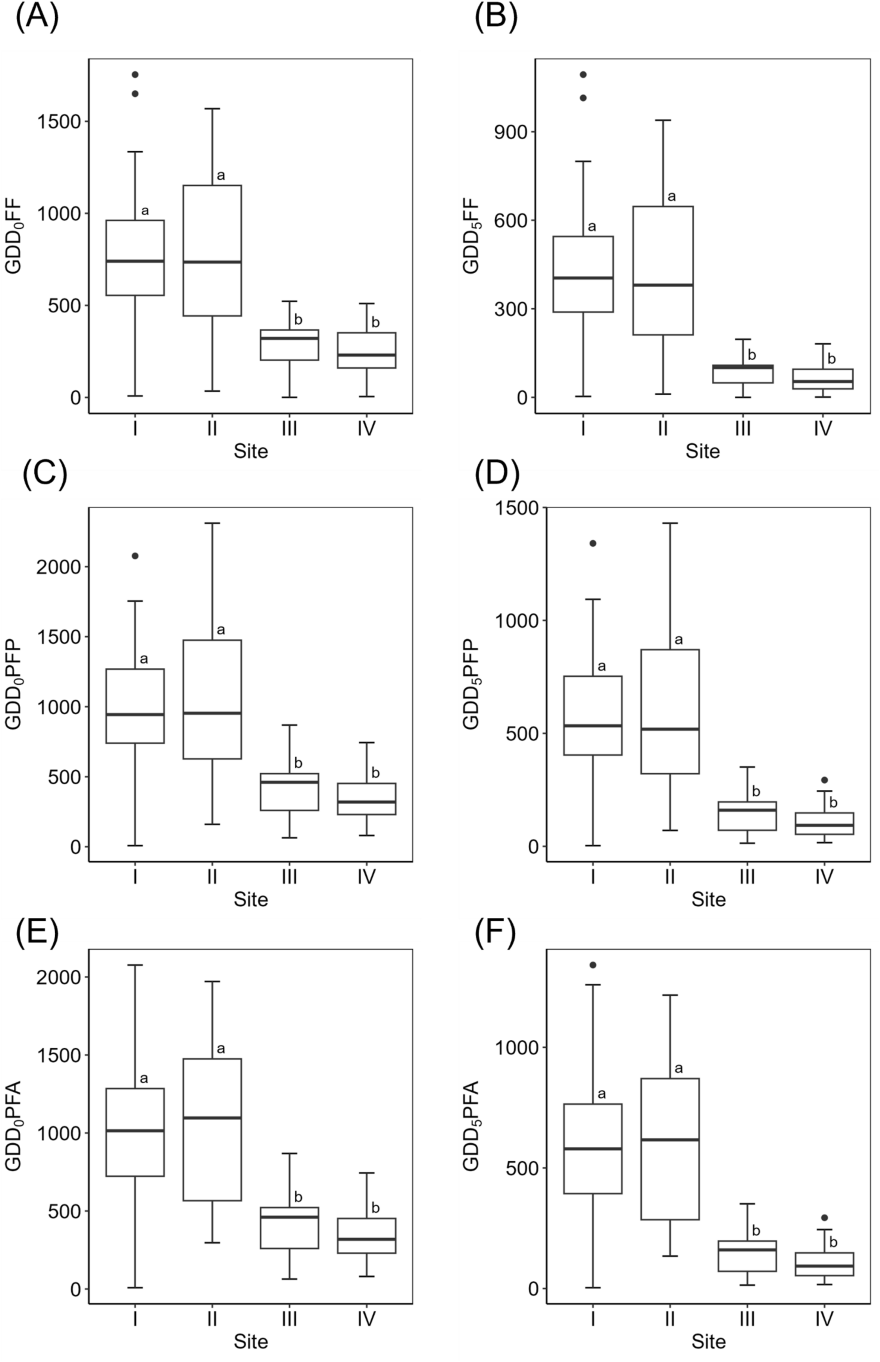
Boxplots of Growing Degree Days (GDD) based phenological traits across four sites. **(A)** GDD for First Flowering at T_BASE_=0 °C; **(B)** GDD for First Flowering at T_BASE_=5 °C; **(C)** GDD for Peak Flowering Plants at T_BASE_=0 °C; **(D)** GDD for Peak Flowering Plants at T_BASE_=5 °C; **(E)** GDD for Peak Floral Abundance at T_BASE_=0 °C and **(F)** GDD for Peak Floral Abundance at T_BASE_=5 °C. Significant differences between sites based on Tukey test or Wilcoxon test are indicated in lower case letters.

While the length of the flowering period per species did not differ significantly among sites (Kruskal-Wallis, χ^2^ = 1.79, p-value=0.62, **Figure 2A**), calendar day to first flowering was significantly earlier in Site I than in the high alpine sites (**Figure 2B**). First flowering (FF), peak flowering plants (PFP) and peak floral abundance (PFA) were significantly shorter in at least one of the high alpine sites than in at least one of the subalpine sites (**Figures 2C–E**). When translated to growing degree days (GDD), high alpine sites always showed significantly lower GDD for FF, PPF and PFA than the subalpine sites (**Figure 3**). Site IV showed significantly greater flowering synchronicity than all other sites (**Figure 2F**).

### 3.3 Functional structure along the elevational gradient

The first three principal components from the PCA analysis were retained to use as orthogonal proxies of trait variation for the functional structure analysis. These components explained 78.6%, 10% and 9.6% of the total trait variance (98.2% overall). PC1 loaded strongly on all GDD-based and most of the calendar-based traits, PC2 was associated with calendar day of first flowering (FF_DOY_) and flowering season length (FSL), and PC3 was dominated by FLS (**Supplementary Table 3**).

While subalpine sites show higher raw values of functional richness (FRic), all four communities exhibited significantly lower FRic than expected under the null model (**Table 2**). Both functional diversity (FDis) and Rao’s quadratic entropy (RaoQ) were markedly lower for the two high alpine compared to the subalpine sites, particularly at Site IV (**Table 2**), indicating that species on the high alpine sites are more functionally similar than on the subalpine sites. In contrast, Site II displayed higher FDis and RaoQ values which do not significantly differ from random expectations, suggesting a lack of trait convergence on that subalpine site.

**Table 2:** Observed values of Functional Richness (FRic), Functional Diversity (FDis) and Rao’s Quadratic Entropy (RaoQ) in each site and standardized effect size of them respect to null communities and associated p-values.

Site	FRic			FDis			RaoQ		
	Obs	SES	p	Obs	SES	p	Obs	SES	p
I	**4.93**	**-4.81**	**<0.001**	**1.32**	**-3.77**	**<0.001**	**2.3**	**-2.23**	**0.02**
II	**5.01**	**-4.41**	**<0.001**	1.46	-1.78	0.08	2.58	-1.1	0.28
III	**1.15**	**-3.81**	**<0.001**	**1.17**	**-3.55**	**<0.001**	**1.59**	**-2.98**	**<0.001**
IV	**1.27**	**-4.21**	**<0.001**	**1.02**	**-5.26**	**<0.001**	**1.21**	**-4.24**	**<0.001**

In bold, value of the index, SES and p when significant.

### 3.4 Phylogenetic signal on phenological traits

When sites were analyzed individually, phylogenetic signal was predominantly detected in flowering times and thermal requirements at the lower elevation sites. At Site I, significant phylogenetic signal was found for traits related to the timing of peak flowering (PFP and PFA), as well as for the growing degree day requirements (all GDD_0_-based traits), whereas at Site II, significant phylogenetic signal also extended to the onset of flowering (FF_DOY_, FF, PFP, PFA and GDD_0_FF; **Table 3**). In contrast, significant phylogenetic signal was largely absent in the high alpine sites, with the exception of flowering season length (FSL) at Site IV (**Table 3**). For all traits and sites, the Ornstein–Uhlenbeck was the best evolution model (**Supplementary Table 4**).

**Table 3:** Values of Blomberg’s K for phylogenetic signal and associated p-value of the five calendar-based flowering traits.

Trait	Site I		Site II		Site III		Site IV	
	K	p-value	K	p-value	K	p-value	K	p-value
FF_DOY_	0.253	0.054	**0.143**	**0.001**	0.358	0.091	0.364	0.082
FF	0.251	0.062	**0.143**	**0.001**	0.358	0.093	0.383	0.073
PFP	**0.316**	**0.014**	**0.074**	**0.031**	0.328	0.146	0.273	0.260
PFA	**0.459**	**0.001**	**0.079**	**0.022**	0.351	0.106	0.345	0.116
FSL	0.238	0.098	0.030	0.511	0.176	0.602	**0.539**	**0.010**
GDD_0_FF	**0.276**	**0.035**	**0.134**	**0.002**	0.295	0.173	0.307	0.173
GDD_0_PFP	**0.308**	**0.013**	0.062	0.069	0.286	0.233	0.271	0.271
GDD_0_PFA	**0.441**	**0.001**	0.062	0.065	0.286	0.227	0.271	0.265

FF_DOY_: Calendar Day to first flowering; FF: days from snowmelt to first flowering; PFP: days from snowmelt to maximum number of plants in flower; PFA: days from snowmelt to maximum number of open flowers; FSL: flowering season length; GDD_0_FF: Growing Degree Days for FF at T_BASE_=0 °C; GDD_0_PFP: Growing Degree Days for PFP at T_BASE_=0 °C; GDD_0_PFA: Growing Degree Days for PFA at T_BASE_=0 °C.

In bold, values K and their associated p-values when significant.

### 3.5 Richness effect assessment and phylogenetic signal

The simulated models of decreasing richness on each site detected a very clear effect of species richness on phylogenetic signal for the GDD metrics; i.e. significant phylogenetic signal is less likely to be detected in the same community as species richness decreases (**Supplementary Figure 3**). As mentioned earlier, to discard that the lack of significant phylogenetic signal in the high alpine sites was related to the lower number of species there, the data from sites belonging to the same altitudinal belt were combined to obtain higher richness levels. The newly calculated flowering traits continued to show significant phylogenetic significance only in the subalpine belt (**Supplementary Table 5**). Therefore, despite the possibility of an effect of differences in species numbers for detecting phylogenetic signal, the lower number of species on the high alpine sites can be confidently dismissed as the reason for a lack of phylogenetic signal in our study system.

### 3.6 Phylogenetic structure of alpine communities

When compared to null models for the same richness, the two high alpine sites showed significantly negative standardizes mean nearest taxon distance (MNTD_SES_) values while for Sites I and II there were no significant effects (**Table 4**). The results for the high alpine sites indicate phylogenetic clustering (i.e. less phylogenetic distance among co-occurring species than expected by chance). However, for standardized mean pairwise distance (MPD_SES_), the values for the two high sites were not significant. Overall, results indicate that phylogenetic clustering is restricted to the high alpine sites.

**Table 4:** Observed values of Mean Pairwise Distance (MPD) and Mean Nearest Taxon Distance (MNTD) in each site, standardized effect size of MPD and MNTD respect to null communities (MPDSES and MNTDSES) and associated p-values.

Site	MPD	MPD_SES_	p-value	MNTD	MNTD_SES_	p-value
I	1.048	0.086	0.544	0.384	0.337	0.634
II	1.100	0.385	0.653	0.311	-0.650	0.291
III	0.749	-1.179	0.094	**0.228**	**-1.680**	**0.014**
IV	0.741	-1.336	0.061	**0.228**	**-1.665**	**0.019**

In bold, value of MPD or MNTD, MPDSES and MNTDSES and p when significant.

## 4 Discussion

We considered two sets of alpine communities (subalpine and high alpine) in the Andes of central Chile separated by around 1000 m elevation to better understand the effect of harsh conditions on the evolution of functional traits and the shaping of communities. We posited that community composition and flowering traits at the highest elevations are the result of *in situ* adaptation of descendants of ancestral lineages and not the product of environmental filtering for ancestral lineages preadapted for life at high elevations. In agreement with our original predictions, we found evidence for convergence in flowering traits at the higher elevation accompanied by loss of phylogenetic signal. Terminal phylogenetic clustering was revealed, suggesting coexistence of closely related groups of taxa in high alpine Andean communities.

### 4.1 Variation in phenological traits across elevations

The lower growing degree days values (GDD) on the higher elevation sites support the notion of strong flowering phenological adjustment over the ~1000 m altitudinal gradient in the central Chilean Andes representing less than seven kilometers of lineal distance. Lower GDD values accompanying flowering onset that was retarded by one month, indicate that, in general, flowering was accelerated at higher elevations. These results agree with earlier work in the alpine (Ahmad et al., 2021; Arroyo et al., 1981; Bucher and Römermann, 2020; Meng et al., 2016). A decrease in thermal sums required for flowering over elevation or snow melt gradients has been reported both for populations of the same species and at the community level (Kawai and Kudo, 2011; Bucher et al., 2018; Arroyo et al., 2021). The effect of this speeding up is significant; if plant species on the upper sites had required the same amount of heat to produce open flowers as those in the lower sites (mean GDD_0_PFA=1038.38), peak flowering in the community would have occurred in early April, well past the warmer period of the year. Huelber et al. (2006) found no difference in cumulative energy input to reach flowering in a 1-month snowmelt gradient, but their research took place on a single site covering a 50m elevational gradient, which is much smaller than the elevational gradient considered in our study.

Not only was flowering speeded up after snowmelt, but flowering overlap among species at higher elevation was greater indicating a tendency for convergence in flowering times. This is also supported by functional structure indices measured across elevations. All sites showed negative values of functional richness (FRic_SES_), indicating that communities represent discrete subsets of the functional space. This suggests that the set of phenological traits present on each site is shaped by local conditions. Moreover, functional dispersion and quadratic entropy (FDis_SES_ and RaoQ_SES_) show significantly more negative values at the higher-elevation sites compared to the lower ones, indicating exceptionally low functional dispersion and pairwise dissimilarity among coexisting species, reinforcing the described pattern of convergence. In contrast, the lower-elevation sites have less extreme FDis_SES_ and RaoQ_SES_ values, suggesting that species in these communities occupy a broader range of functional strategies. Taken together, these results highlight the adaptive and evolutionary lability of flowering phenology along the elevational gradient. Schroeder et al. (2024) found that phylogenetic dispersion related to vegetative traits was also lower at 3500 m a.s.l. than at 2400 m a.s.l. in the central Chilean Andes. It is worth noting that the flowering patterns documented in our study were recorded during a single flowering season and therefore reflect the climatic condition of that particular year. Nevertheless, the large temperature differences between the two elevational belts considered are expected to be relatively consistent despite interannual variation. Moreover, previous studies indicate that the study period fell within the normal range of interannual temperature variation for the region (Arroyo et al., 2020).

The two components of phenological adjustment over elevational gradient (faster flowering and greater flowering time overlap) found in this study suggests a strong selective pressure of colder temperatures and perhaps of the length of the flowering season. If phenological adjustment is driven by an evolutionary process governed by temperature favoring trait convergence, a weakening of phylogenetic signal would be observed. Accordingly, phylogenetic signal on floral metrics was almost absent in the high alpine sites yet clearly present on the lower sites. Low Blomberg’s K values may be interpreted as evidence of trait convergence operating at all sites but with much greater strength in the upper sites. Although only slightly, Site I was the warmest of the two subalpine sites, and consequently, temperature would exert less selective pressure. Thus, it is perhaps not surprising that higher phylogenetic signal was found in all GDD-based traits on this site in comparison with the cooler subalpine site II.

Similar weakening in phylogenetic signal at higher elevations for flowering has been reported on other alpine gradients. Although no significant phylogenetic signal was detected by Basnett et al. (2019) in several elevational bands, an elevational decrease in the values of Blomberg’s K and Pagel’s λ was observed across a 1200 m elevation gradient. In the same way, Lessard-Therrien et al. (2014) showed less frequent significant phylogenetic signal at higher elevation over a 200 m elevational gradient. It should be pointed out, however, that these results could be influenced by the differences in species richness over elevation in those systems. Nonetheless, stronger phylogenetic signal at higher elevations was reported by D. Li et al. (2021) in a sea-level to high mountain gradient. These authors invoked the tropical niche conservatism hypothesis to explain that colder regions communities (like high alpine communities) are younger and shaped by environmental filtering, thus, showing similar phenological traits in closely related species.

It is worth mentioning that our phylogenetic signal analyses were conducted on an ecological community rather than on a well-sampled clade. Any community phylogeny represents only a subset of taxa drawn from multiple clades, and, therefore, misses trait information from taxa that are absent in that environment. While this approach may not be ideal for investigating the evolutionary mechanisms that shape traits, it certainly offers valuable insight into eco-evolutionary patterns, such as those underlying community assembly (Caradonna and Inouye, 2015).

### 4.2 Phylogenetic structure of alpine communities

**Figure 4:**
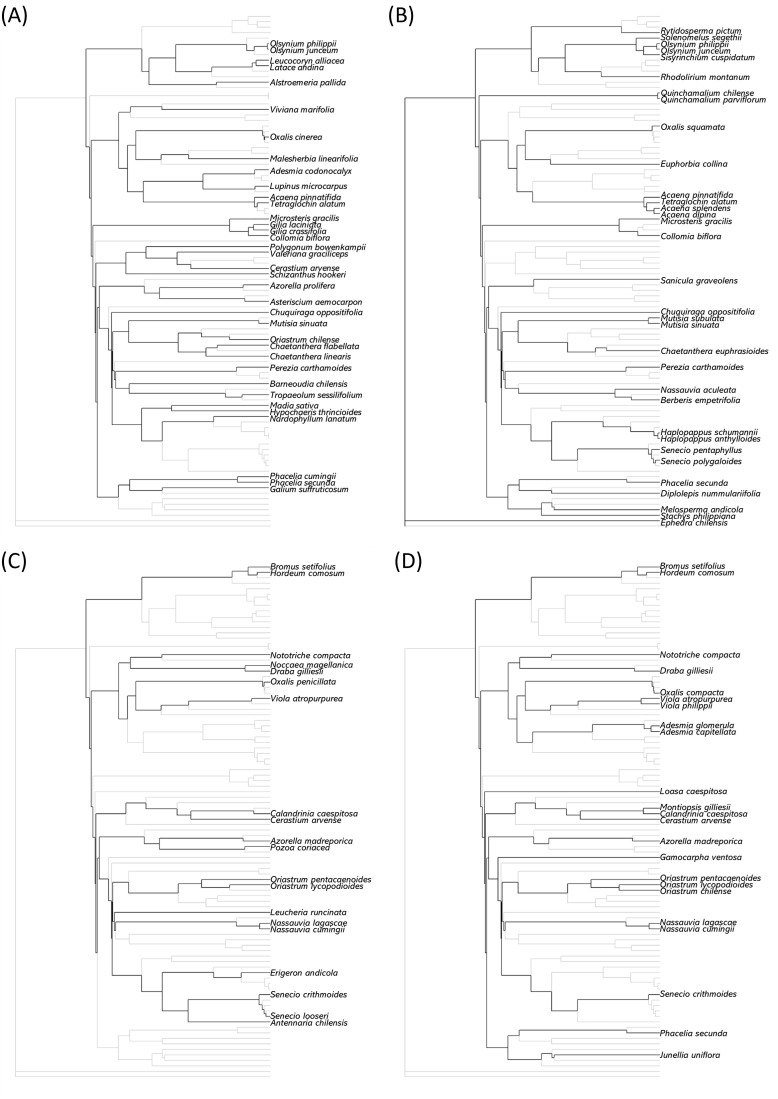
Pruned phylogenies showing the species composition of the four sites: **(A)** Site I, **(B)** Site II, **(C)** Site III, and **(D)** Site IV. Highlighted branches indicate the species present at each site, displayed within the context of the full phylogeny for the study’s species pool (gray background branches).

In our study, we found significant phylogenetic clustering at the higher sites which is commonly interpreted as evidence of environmental filtering (Webb et al., 2002). However, significant values of standardized mean nearest taxon distance (MNTD_SES_) but not for standardized mean pairwise distance (MPD_SES_) indicate that the clustering on these sites is concentrated near the tips of the phylogeny rather than at its base, reflecting the coexistence of many clusters of closely related, relatively recently-evolved taxa spread over several lineages rather than a concentration of closely related species representing a limited number of lineages (**Figure 4**).

In the same venue, a recent review of 73 high elevation communities found a global tendency for phylogenetic clustering which was ascribed to environmental filtering imposed by the cooler temperatures at higher elevations (Qian et al., 2021). But when looking at the elevational gradient, some studies show increasing clustering with elevation (Li et al., 2014; Zhang et al., 2020) while others report the opposite trend (Culmsee and Leuschner, 2013; González-Caro et al., 2014; López-Angulo et al., 2018; Luo et al., 2023) and even increasing clustering followed by increasing dispersion in different altitudinal belts (Li et al., 2022). For our study, we used both the deep-sensitive MPD and the terminal-sensitive MNTD metrics. Analyses based on only one of these metrics run the risk of drawing erroneous conclusions regarding whether the phylogenetic structure of a community is shaped by environmental filtering, limiting similarity or non-restrictive colonization.

Given our phylogenetic structure results and the demonstration of phylogenetic signal weakening, the most likely scenario for the assembly process of these communities is the colonization of lineages dispersed across the phylogeny, with environmental filtering acting over non-clustered traits or not acting at all, followed by small radiations in the newer environment and strong adaptation resulting in convergent flowering traits across distinct species. This scenario reflects the “Tropical Niche Conservatism” hypothesis, in that taxa pertaining to high alpine communities in the central Chilean Andes are the descendants of lineages that managed to shift their niches to colder conditions (Wiens and Donoghue, 2004). Nonetheless, terminal but not basal phylogenetic clustering suggests that colonization to the alpine is not a rare event, recalling the “Out of the Tropics” (OTT) hypothesis (Jablonski et al., 2006). The OTT hypothesis posits that niche conservation, although it exists, does not limit the expansion of taxa into colder environments to the extent that upward colonizing lineages would be randomly distributed in the phylogeny (Qian and Ricklefs, 2016). This pattern is consistent with diversification documented in several Andean genera such as *Adesmia* (Pérez et al., 2024), *Senecio* (Salomón et al., 2025) and *Chaeanthera* (Hershkovitz et al., 2006). Regardless of whether the present-day species in the high alpine belt are a product of upward migration of linages, or dispersal along the Andean corridor, selective pressures and/or environmental filtering to adjust their flowering times would have taken place *in situ* o prior to migration along the Andean corridor, contributing to the observed patterns of functional structure and phylogenetic signal. Overall, our results support that taxa in both elevational belts have been subjected to selective pressures and evolved in these novel environments, but these pressures appear to have been stronger at the high alpine belt, where phylogenetic signal has weakened to a greater extent.

The integration of trait data and a thorough analysis of phylogenetic structure metrics suggest that abiotic factors act more as the drivers of evolution than as gatekeepers in these environments. Finally, if we had adhered faithfully to Webb et al. (2002) and limited our analysis to one of the phylogenetic structure metrics, we would have concluded that environmental filtering has been the main force shaping high alpine community composition. The integration of different analytic approaches suggests the co-occurrence of different processes often seen as non-complementary (i.e. environmental filtering combined with trait convergence). These findings endorse the notion that community assembly is a complex and irreducible process where straightforward and discrete frameworks can be misleading.

### 4.3 Community size and phylogenetic signal

To our mind, an important contribution of our study is the rigorous treatment of the potential effect of sample size on the detection of phylogenetic signal. Basnett et al. (2019) found phylogenetic signal in 10 species of *Rhododendron* distributed across a full mountain gradient but not in the 3 to 5 species per 100 m altitudinal band while Krasnov et al. (2011) found it in 218 species in a continental pool of insects but not the 10 to 39 species of insects per regional pool. These authors concluded that phylogenetic signal is geographically and taxonomically scale-dependent. However, these results could also reflect an effect of sample size on phylogenetic signal as per shown in the theoretical work of Münkemüller et al. (2012). Here we introduced a method to detect this potential problem in studies where species numbers are unbalanced, as occurs over the alpine gradient and, indeed, in community comparisons in general. Effectively, in this study, we confirmed the decreasing probability of detecting phylogenetic signal with decreasing richness. To overcome this potential problem, we then merged sites belonging to the same altitudinal belt to obtain a species richness value similar to that in the lower-elevation sites. Upon controlling for species richness, phylogenetic signal persisted in the subalpine sites while it remained absent in the high alpine community. Consequently, we feel confident that our interpretation that phylogenetic signal on flowering traits becomes lost at the higher elevations and adaptation overrides phylogenetic conservatism is not a spurious result.

## 5 Conclusion

Our results show that high alpine communities in the South American Andes in the central Chilean Andes have undergone significant flowering phenological adjustment allowing them to flower more rapidly after snowmelt and under colder conditions than their subalpine counterparts, leading to lower levels of phylogenetic signal for flowering phenology traits compared with the latter. This pattern suggests adaptive convergence in functional trait among these mediterranean-type climate alpine plant communities as a response to the harsher and hence more challenging environmental conditions prevalent at very high elevations. Significant decreases in thermal sums required for flowering and loss of phylogenetic signal at the higher elevation sites suggest that temperature and the shortening of the growing season are likely among the main drivers of these changes. Values of MPD_SES_ and MNTD_SES_ indicate that the high alpine communities are phylogenetically clustered towards the terminal portion of the tree. Thus, environmental filtering for preadapted lineages appears to be less important than is *in situ* adaptation in the conformation of the studied high elevation communities. A combination of non-restrictive expansion of lineages towards higher elevations, strong selective pressures and convergent evolution on functional traits appears to have shaped high alpine communities in the Chilean central Andes. In summary, abiotic factors may act more as the drivers of evolution than gatekeepers in these environments. To avoid spurious results, we strongly recommend that the effect of sample size on the detection of phylogenetic signal be considered in ecological studies.

## Data Availability

The datasets presented in this study are available in online repositories. The phenology data and phylogenetic tree are available in Mendeley Data (doi: 10.17632/dhjdww5xsj.1), and the genetic sequence accession numbers are provided in the **Supplementary Material**.
