# Supplementary material for: Phylogenetic signal in flowering phenology weakens over elevation in the high Andes of Chile: evidence for evolutionary convergence in a harsh habitat
Source: Front Plant Sci. 2026 Feb 17;17:1738754. doi: 10.3389/fpls.2026.1738754 (PMC12962001; doi:10.3389/fpls.2026.1738754)
Supplement: Supplementary file 1 [file DataSheet1.pdf]

Supplementary Material

1 Supplementary Data

1.1 DNA extraction, amplification, and sequencing

Genomic DNA was extracted with the DNeasy Plant Kit (Qiagen, Valencia, CA, USA). We amplified the DNA using the primers listed in Table 1. PCR used a final volume of 30 µl, which contained 4 µl DNA (25 ng/µl), 8.35 µl distilled water, 3 µl MgCl2 (25 mM), 6 µl buffer, 2.4 µl of dNTP (1 mM), 1.8 µl of each primer (10x), 2.4 µl BSA (25 mM) and 0.25 µl GoTaq (5 U/ µl). For ITS, DNA was denatured at 95°C for 5 min, followed by 35 amplification cycles of 45 s at 94°C, annealing for 1 min at 50°C, elongation for 1.5 min at 72°C and a final extension of 7 min at 72°C. For *rbc*L, DNA was denatured at 95°C for 3 min. Followed by 30 amplification cycles of 1 min at 95°C, annealing for 45 s at 50°C, elongation for 1 min at 65°C and a final extension of 5 min at 65°C, and *matK*, DNA was denatured at 95°C for 3 min. Followed by 36 amplification cycles of 30 s at 94°C, annealing for 40 s at 58°C, elongation for 1 min at 72°C and a final extension of 10 min at 72°C. Samples were sent to Macrogen (Seoul, South Korea) for purification and sequencing. Sequences were loaded, edited and aligned using ChromasPro 2.33 (Technelysium, Brisbane, Australia) and BioEdit 7.0 (Hall, 1999) and have been deposited in GenBank (Table S1).

Supplementary Table 1. Herbarium voucher numbers and GenBank accession numbers for ITS, *rbc*L and *mat*K for all species used in the phylogeny.

Species	Herbarium voucher	ITS	rbcL	matK
Acaena alpina	CONC 184996	MH781148	ON542515	ON542548
Acaena pinnatifida	CONC 185086	MH781149	MF963399	MF963761
Acaena splendens	CONC 184995	MH781150	ON542549	ON542516
Adesmia capitellata	CONC 185061	MH781152	MZ198420	To be uploaded
Adesmia codonocalyx	CONC 185068	MH781153	To be uploaded	To be uploaded
Adesmia glomerula	CONC 185073	MH781156	MZ198417	To be uploaded
Alstroemeria pallida	Obtained from GenBank	EU159930	JQ404687	EU159956
Antennaria chilensis	CONC 190331	To be uploaded	To be uploaded	To be uploaded
Asteriscium aemocarpon	CONC 185088	MH781162	To be uploaded	To be uploaded
Azorella madreporica	CONC 185063	MH781165	MZ198403	ON542519
Azorella prolifera	CONC 190411	To be uploaded	To be uploaded	To be uploaded
Barneoudia chilensis	Obtained from GenBank	FJ639901	FJ639865	Not used
Berberis empetrifolia	CONC 184994	MH781167	MZ198467	Not used
Bromus setifolius	CONC 194310	To be uploaded	To be uploaded	To be uploaded
Calandrinia caespitosa	Obtained from GenBank	DQ090319	To be uploaded	To be uploaded
Cerastium arvense	CONC 184989	MH781177	ON542552	ON542522
Chaetanthera euphrasioides	Obtained from GenBank	DQ355866	ON542553	ON542523
Chaetanthera flabellata	Obtained from GenBank	DQ355867	To be uploaded	To be uploaded
Chaetanthera linearis	CONC 185010	MH781179	To be uploaded	To be uploaded
Chuquiraga oppositifolia	Obtained from GenBank	EU841151	EU841109	EU841332
Collomia biflora	CONC 185087	MH781180	ON542554	HQ116935
Diplolepis nummulariifolia	CONC 190284	To be uploaded	To be uploaded	To be uploaded
Draba gilliesii	CONC 185033	MH781187	MZ198441	To be uploaded
Ephedra chilensis	Obtained from GenBank	Not used	AY492036	AY492012
Erigeron andicola	CONC 185093	MH781190	MZ198374	To be uploaded
Euphorbia collina	CONC 190283	To be uploaded	ON542555	Not used
Galium suffruticosum	CONC 190442	To be uploaded	To be uploaded	To be uploaded
Gamocarpha ventosa	CONC 185039	MH781225	To be uploaded	To be uploaded
Gilia crassifolia	CONC 185043	MH781199	To be uploaded	To be uploaded
Gilia laciniata	CONC 184984	MH781200	To be uploaded	EU628540
Gingko biloba	Obtained from GenBank	EF372233	AJ235804	EF468640

Supplementary Table 1. Cont.

Species	Herbarium voucher	ITS	rbcL	matK
Haplopappus anthylloides	CONC 185055	MH781202	ON542556	ON542524
Haplopappus schumannii	CONC 185092	MH781204	ON542557	ON542525
Hordeum comosum	Obtained from GenBank	AJ607876	AY137441	AB078097
Hypochaeris thrincioides	CONC 190266	To be uploaded	To be uploaded	To be uploaded
Junellia uniflora	CONC 194305	To be uploaded	To be uploaded	To be uploaded
Latace andina	Obtained from GenBank	KF171082	Not used	Not used
Leucheria runcinata	CONC 29063A	KY010374	Not used	To be uploaded
Leucocoryne alliacea	CONC 194308	KF171086	Not used	To be uploaded
Loasa caespitosa	CONC 194327	To be uploaded	To be uploaded	To be uploaded
Lupinus microcarpus	CONC 185006	MH781211	MG247571	To be uploaded
Madia sativa	CONC 185046	MH781212	AY215141	To be uploaded
Malesherbia linearifolia	CONC 185009	MH781213	AF206792	EF135562
Melosperma andicola	CONC 185014	MH781214	MZ198432	AY492153
Microsteris gracilis	CONC 194982	MH781217	To be uploaded	To be uploaded
Montiopsis gilliesii	CONC 28904	DQ090406	To be uploaded	HQ620880
Mutisia sinuata	CONC 185048	MH781221	EU841128	EU841355
Mutisia subulata	CONC 185076	MH781222	To be uploaded	To be uploaded
Nardophyllum lanatum	CONC 185044	MH781223	To be uploaded	To be uploaded
Nassauvia aculeata	CONC 190315	MG432159	MZ198387	To be uploaded
Nassauvia cumingii	CONC 185057	MG432161	To be uploaded	To be uploaded
Nassauvia lagascae	CONC 194309	MG432166	To be uploaded	To be uploaded
Noccaea magellanica	CONC 185067	MH781228	MZ198437	To be uploaded
Nototriche compacta	CONC 185074	MH781229	To be uploaded	To be uploaded
Olsynium junceum	CONC 29044	JN389210	To be uploaded	To be uploaded
Olsynium philippii	Obtained from GenBank	JN389212	Not used	JN565608
Oriastrum chilense	CONC 25180	DQ355916	To be uploaded	To be uploaded
Oriastrum lycopodioides	CONC 29084	DQ355920	MZ198388	To be uploaded
Oriastrum pentacaenoides	CONC 29077A	DQ355904	To be uploaded	To be uploaded
Oxalis cinerea	Obtained from GenBank	JN634728	Not used	Not used
Oxalis compacta	CONC 185002	MH781235	To be uploaded	To be uploaded
Oxalis penicillata	CONC 185035	MH781236	JN587336	To be uploaded
Oxalis squamata	CONC 185011	MH781237	JN587339	To be uploaded
Perezia carthamoides	CONC 194307	FJ979641	EU841130	To be uploaded
Phacelia cumingii	CONC 190447	To be uploaded	To be uploaded	To be uploaded

Supplementary Table 1. Cont.

**Species**	**Herbarium voucher**	**ITS**	**rbcL**	**matK**
Phacelia secunda	CONC 184998	ON521132	ON542564	ON542536
Polygonum bowenkampii	CONC 185069	MH781241	Not used	ON542537
Pozoa coriacea	CONC 185016	To be uploaded	ON542565	ON542538
Quinchamalium chilense	CONC 185078	MH781243	Not used	Not used
Quinchamalium parviflorum	CONC 194352	MH781244	Not used	Not used
Rhodolirium montanum	CONC 184997	MH781245	Not used	ON542539
Rytidosperma pictum	Obtained from GenBank	GU359227	EU400674	EU400765
Sanicula graveolens	CONC 194325	ON521133	MF963118	ON542540
Schizanthus hookeri	CONC 185101	MH781247	Not used	EF439052
Senecio crithmoides	CONC 190327	EF538321	MZ198397	To be uploaded
Senecio looseri	CONC 185036	MH781251	To be uploaded	To be uploaded
Senecio pentaphyllus	CONC 185029	MH781252	ON542567	ON542542
Senecio polygaloides	CONC 185028	MH781253	To be uploaded	To be uploaded
Sisyrinchium cuspidatum	CONC 185023	MH781256	ON542569	ON542544
Solenomelus segethii	Obtained from GenBank	JN389266	JQ670577	JX903637
Stachys philippiana	CONC 185100	MH781259	ON542570	ON542545
Tetraglochin alatum	CONC 185051	MH781260	ON542571	ON542546
Tropaeolum sessilifolium	CONC 194303	DQ007282	To be uploaded	To be uploaded
Valeriana graciliceps	Obtained from GenBank	HQ878137	Not used	JF269473
Viola atropurpurea	CONC 185060	MH781264	ON542572	To be uploaded
Viola philippii	CONC 185015	MH792062	ON542573	To be uploaded
Viviania marifolia	CONC 185080	MH781266	L14707	To be uploaded

Supplementary Table 2. Primers used for DNA amplification.

Primer	Sequence (5´-3´)	References
ITS4	TCCTCCGCTTATTGATATGC	White et al. 1990
ITS5	GGAAGTAAAAGTCGTAACAAGG	White et al. 1990
*rbc*L 1F	ATGTCACCACAAACAGAAACTAAAGCA	Olmstead et al. 1992
*rbc*L 1460R	TCCTTTTAGTAAAAGATTGGGCCGAG	Olmstead et al. 1992
matK KIM 1R	ACCCAGTCCATCTGGAAATCTTGGTTC	Kim KJ 2009, unpublished
matK KIM 3F	CGTACAGTACTTTTGTGTTTACGAG	Kim KJ 2009, unpublished

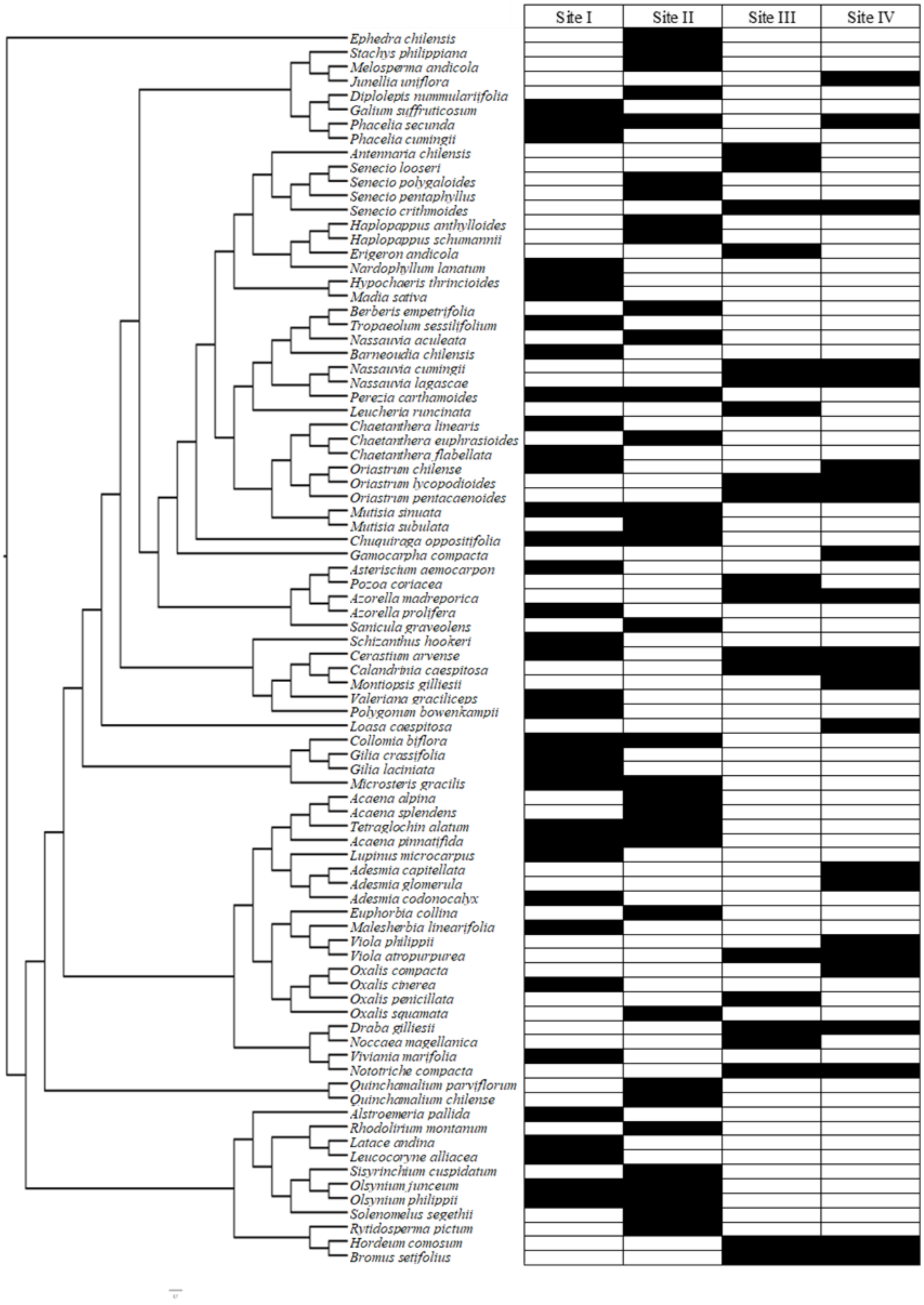

Supplementary Figure 1. Phylogenetic tree of all study area species based on Bayesian inference and local presence of species in each site.

Supplementary Table 3. Trait loadings on the first three principal components (PCs) obtained from a PCA of standardized flowering traits for the species included in this study.

Trait	PC1	PC2	PC3
FF_{DOY}	0.168	-0.721	-0.395
PFP	0.327	-0.197	-0.029
PFA	0.323	-0.244	0.037
FSL	0.043	-0.415	0.866
FF	0.323	-0.162	-0.203
GDD_0FF	0.333	0.147	-0.079
GDD_5FF	0.327	0.234	-0.039
GDD_0PFP	0.334	0.112	0.065
GDD_5PFP	0.328	0.190	0.089
GDD_0PFA	0.332	0.128	0.114
GDD_5PFA	0.326	0.204	0.132

Supplementary Table 4. Values of the Akaike Information Criterion (AIC) of Brownian motion (BM), Ornstein–Uhlenbeck (OU), and early-burst (EB) evolution models for each trait in each site. In bold, lowest value of AIC for each trait in each site.

	Site I			Site II			Site III			Site IV		
	AIC_{BM}	AIC_{OU}	AIC_{EB}	AIC_{BM}	AIC_{OU}	AIC_{EB}	AIC_{BM}	AIC_{OU}	AIC_{EB}	AIC_{BM}	AIC_{OU}	AIC_{EB}
FF_{DOY}	362.3	**353.6**	363.9	332.5	**322.6**	335.0	191.8	**188.6**	194.6	217.3	**213.7**	220.0
PFP	358.4	**352.2**	360.5	363.4	**348.7**	365.8	196.0	**192.5**	198.8	224.4	**218.8**	227.0
PFA	347.4	**345.7**	349.6	354.1	**339.8**	356.5	196.0	**192.5**	198.8	215.7	**211.7**	218.4
FSL	361.7	**351.7**	363.1	366.2	**348.5**	368.6	209.5	**202.9**	212.3	206.8	**206.2**	209.4
FF	362.3	**353.6**	363.9	332.5	**322.6**	335.0	191.8	**188.6**	194.6	215.3	**211.6**	217.9
GDD_0FF	543.7	**535.7**	545.4	501.3	**490.7**	503.7	271.4	**267.3**	274.2	304.3	**299.5**	307.0
GDD_5FF	508.7	**501.1**	510.5	470.3	**459.3**	472.7	236.4	**231.5**	239.2	262.8	**257.2**	265.5
GDD_0PFP	550.0	**542.6**	552.1	542.7	**527.3**	545.2	280.2	**276.0**	283.0	319.1	**313.3**	321.7
GDD_5PFP	520.0	**512.2**	522.2	516.6	**500.8**	519.0	248.7	**244.1**	251.5	282.7	**276.8**	285.3
GDD_0PFA	541.4	**538.2**	543.5	536.2	**520.8**	538.6	280.2	**276.0**	283.0	319.1	**313.3**	321.7
GDD_5PFA	512.6	**508.9**	514.7	511.6	**495.8**	514.0	248.7	**244.1**	251.5	282.7	**276.8**	285.3

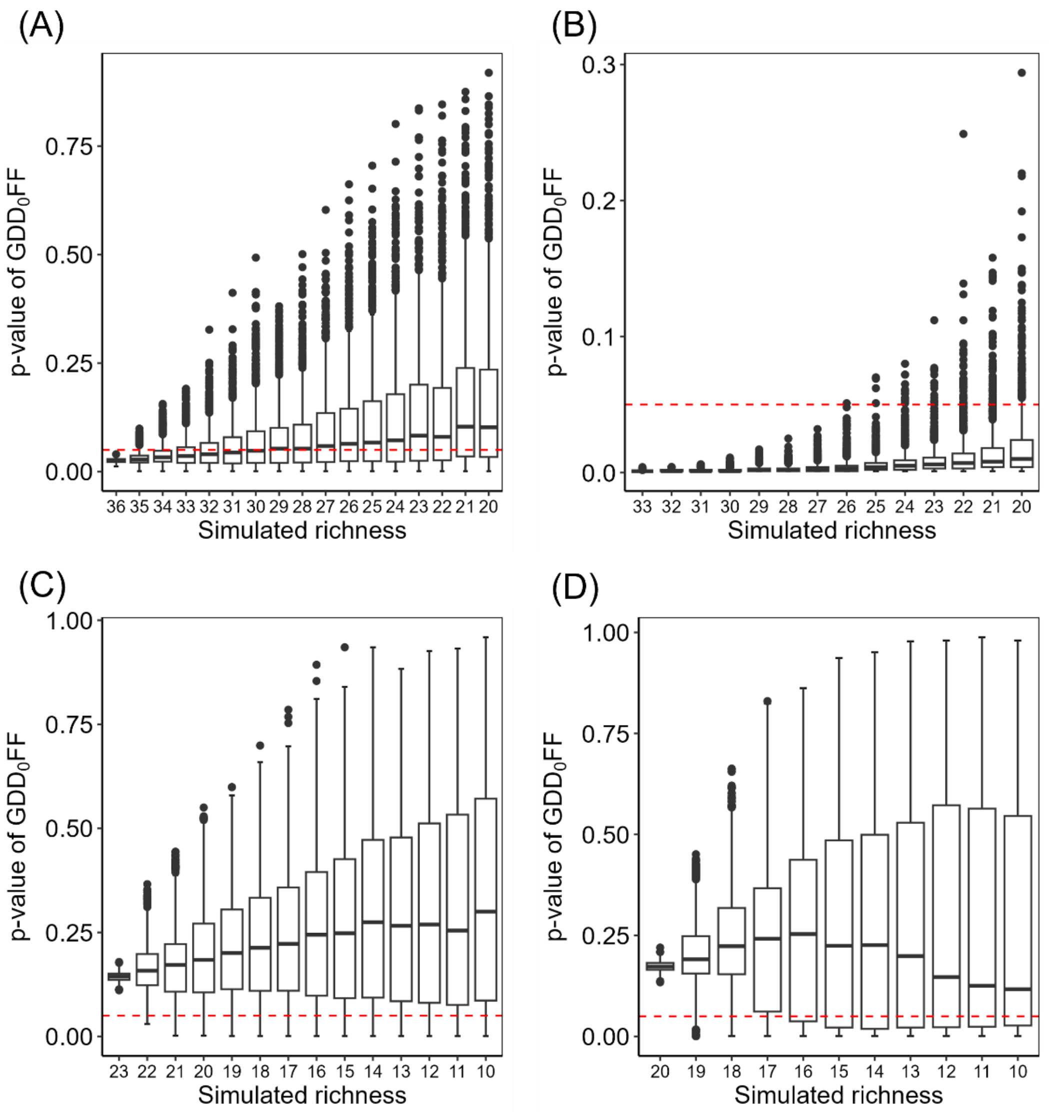

Supplementary Figure 3. Example of distribution of p-values for Blomberg's K randomizations under subsequential richness subsampling for each site in GDD_0FF. In each site (Sites I, II, III and IV corresponding to panels A, B, C and D respectively), richness starts at original value and decreases to 20 species in subalpine sites and to 10 species in high alpine sites. A dotted red line indicates a significance level of 0.05.

Supplementary Table 5. Values of Blomberg's K for phylogenetic signal and associated p-value of floral metrics for the artificial communities from joined data of each altitudinal belt. In bold, value of K and p when significant.

	Subalpine		High alpine	
	K	p-value	K	p-value
FF_{DOY}	**0.111**	**0.006**	**0.313**	**0.034**
FF	**0.090**	**0.026**	0.236	0.140
PFP	**0.100**	**0.012**	**0.299**	**0.042**
PFA	0.054	0.278	0.205	0.227
FSL	**0.114**	**0.006**	**0.316**	**0.028**
GDD_0FF	**0.110**	**0.007**	0.239	0.119
GDD_5FF	**0.107**	**0.009**	0.223	0.164
GDD_0PFP	**0.083**	**0.039**	0.215	0.193
GDD_5PFP	**0.080**	**0.047**	0.208	0.211
GDD_0PFA	**0.084**	**0.032**	0.213	0.198
GDD_5PFA	**0.078**	**0.051**	0.203	0.238

FF_{DOY}: Calendar Day to first flowering; FF: days from snowmelt to first flowering; PFP: days from snowmelt to maximum number of plants in flower; PFA: days from snowmelt to maximum number of open flowers; FSL: flowering season length. GDD_0FF: GDD for FF at T_{BASE}=0°C; GDD_5FF: GDD for FF at T_{BASE}=5°C; GDD_0PFP: GDD for PFP at T_{BASE}=0°C; GDD_5PFP: GDD for PFP at T_{BASE}=5°C; GDD_0PFA: GDD for PFA at T_{BASE}=0°C; GDD_5PFA: GDD for PFA at T_{BASE}=5°C.
